# Effect of a family focused active play intervention on sedentary time and physical activity in preschool children

**DOI:** 10.1186/1479-5868-9-117

**Published:** 2012-10-01

**Authors:** Mareesa V O’Dwyer, Stuart J Fairclough, Zoe Knowles, Gareth Stratton

**Affiliations:** 1 Early Childhood Ireland, Hainault House, Belgard Square, Tallaght, Dublin 24, Ireland; 2 Research Institute of Sports and Exercise Science, Liverpool John Moores University, Tom Reilly Building, Byrom Street, Liverpool, L3 3AF, UK; 3 Research Centre for Sports and Exercise Sciences, College of Engineering, Swansea University SA2 8PP, Swansea, UK

**Keywords:** Preschool children, Parent involvement, Active play, Physical activity, Sedentary time, Accelerometry, Intervention, Multi-level analysis

## Abstract

**Background:**

Early childhood provides a window of opportunity for the promotion of physical activity. Given the limited effectiveness of interventions to date, new approaches are needed. Socio-ecological models suggest that involving parents as intervention targets may be effective in fostering healthier lifestyles in children. This study describes the effectiveness of a family-focused ‘Active Play’ intervention in decreasing sedentary time and increasing total physical activity in preschool children.

**Method:**

Seventy-seven families were recruited from 8 randomly selected SureStart children’s centres in the North West of England. Centres were randomly assigned to either an intervention (n = 4) or a comparison group (n = 4). Parents and children in the intervention group received a 10-week active play programme delivered by trained active play professionals; this included an activity and educational component. Families in the comparison group were asked to maintain their usual routine. Each participating parent and child wore a uni-axial accelerometer for 7 days at baseline and post-test. Week and weekend day sedentary time and total physical activity adjusted for child- and home- level covariates were analysed using multilevel analyses.

**Results:**

Significant intervention effects were observed for sedentary time and physical activity for both week and weekend days. Children in the intervention group engaged in 1.5% and 4.3% less sedentary time during week and weekend days, respectively and 4.5% and 13.1% more physical activity during week and weekend days, respectively than children in the comparison group. Parent’s participation in sport and their physical activity levels, child’s sex, availability of media in the home and attendance at organised activities were significant predictors of sedentary time and physical activity in this age group.

**Conclusion:**

A 10-week family focused active play intervention produced positive changes in sedentary time and total physical activity levels in preschool children. Specific covariates were identified as having a significant effect on the outcome measures. Moreover, children whose parents were active engaged in less sedentary time and more physical activity suggesting that parent’s activity habits are mediators of physical activity engagement in this age group.

## Background

Physical activity and sedentary behaviour in early childhood have significant effects on health parameters. Sufficiently active preschool children have increased protection against obesity [1] and cardiovascular disease [2]. Physical activity during the preschool day and limited outdoor playtime are related to body mass index (BMI) in young children [3,4]. Also insufficient physical activity can have a negative impact on psychosocial factors such as self-esteem [5] and are associated with poor fundamental movement skill acquisition during childhood [6]. The early years are an ideal window to promote physical activity, as motor development at this life stage is more malleable than in later childhood and adolescence [7,8], and risk factors for overweight can be more easily modified [9]. Furthermore physical activity levels during the early years of childhood are predictive of activity levels later in adulthood [10]. Despite the evidence supporting the benefits of physical activity during the formative years of life, preschool children do not engage in enough physical activity during the weekdays and weekend days [11,12] and additionally accumulate high levels of sedentary time during these parts of the week [13]. Additionally, studies have shown that preschool children’s physical activity differ on weekdays and weekend days and further research is warranted to decipher the reason why this may be [14].

Studies investigating the correlates of physical activity in children have found parent attitudes, behaviours, parenting styles and practices to have a profound influence on children’s health behaviours [2,15,16]. For example, one study [2] found that children whose parents received information on how, when, and where to encourage their child’s physical activity, spent more time playing outdoors in comparison to children whose parents received no information. Additionally, studies investigating the correlates of sedentary behaviour in this age group have reported indeterminate associations between variables such as television viewing, age, gender and BMI, however a significant negative association between parental rules and sedentary behaviours was reported [17].

There is a need to explore both feasibility and efficacy of parent targeted lifestyle interventions that aim to influence the health behaviours of children. For such interventions to be effective, the active involvement of parents is particularly important [18]. Interventions have previously been conducted where parents contribute in a low to medium capacity e.g. consenting to participation, through home tasks, or receiving letters [2,19,20]. According to De Bock and colleagues [21], the effects of directly exposing parents to an intervention have been understudied yet parents’ participation in interventions is essential given the evidence to suggest significant correlations that exist between parental support and child physical activity level [22]. Parents play a vital role in the facilitation of their child’s physical activity. They are knowledgeable about the barriers to physical activity and have a sense for opportunities that are consistent with their child’s preferences [23]. Furthermore, parental behaviour is noted as one of the strongest determinants of both child physical activity [24] and BMI [25,26]. They can provide an environment which affords their children playful opportunities, allowing them to practice different motor activities and improve their skills [27]. The role of parents within a physical activity intervention may therefore foster more active lifestyles during the preschool years and beyond. However, few interventions targeting preschool children have investigated the effectiveness of directly involving parents within physical activity interventions and little is known about how to successfully engage and motivate parents and other caregivers to promote and support children’s physical activity at home. Moreover, the evidence related to physical activity interventions in child care settings is not definitive and given that parents play a significant role in shaping and supporting their children’s physical activity behaviour further research is warranted regarding their involvement [15,28]. Due to the limited intervention based research targeting child care settings, parents must be willing to take responsibility for encouraging and supporting their children’s physical activity behaviour. Consequently, the development of programs to educate and support parents in this endeavour should be a priority.

Therefore, the aims of this study are first to investigate the effect of a family focused “Active Play” intervention on children’s weekday and weekend day sedentary time and total physical activity, and second to investigate the influence of mediating and moderating variables on sedentary time and total physical activity.

## Methods

### Participants and settings

Twenty-four SureStart children’s centres from a large city in the North West of England were invited to take part in this study. SureStart children’s centres are a free service for families with children aged 5 years or under and are situated in the most disadvantaged parts of England. They provide a variety of advice and support for parents/carers and services are targeted from pregnancy through to entry into compulsory education [29]. All children’s centres were located in neighbourhoods in the highest 10% for national deprivation [30]. Of the 24 children’s centres invited, 15 agreed and 8 were randomly selected to take part in the study.

**Figure 1:**
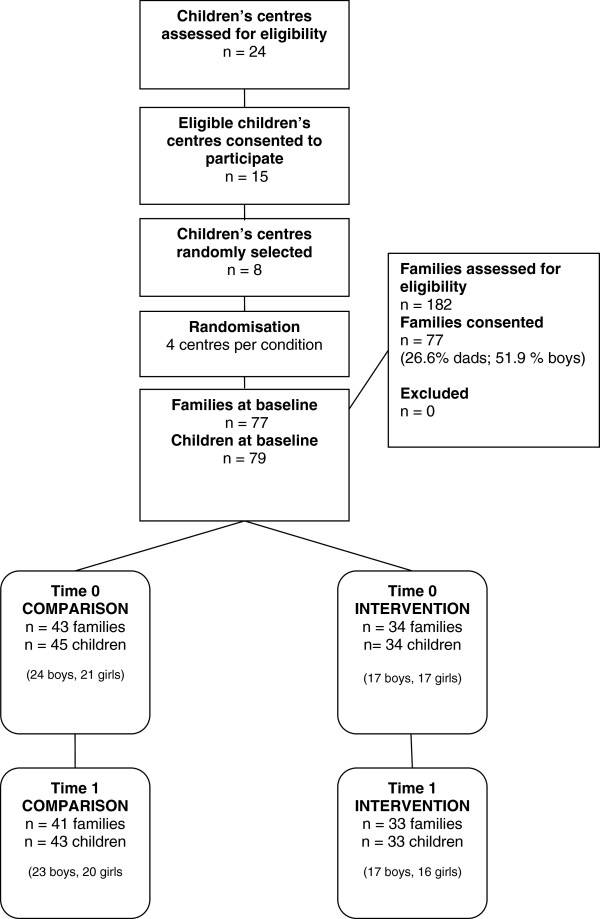
**Children centre and flowof families through theproject.**

Initially, the research team organised a meeting with a member of staff from the children’s centre, typically a health promotion worker or alternate professional. The aim of this meeting was to describe the project and outline the aims of the research. The children’s centre staff received information packs and distributed them to eligible families. Information packs contained a participant information letter, consent form, assent form, medical questionnaire and preschool-age physical activity questionnaire (Pre-PAQ) [31]. To be eligible to take part children had to be registered at the participating children’s centre, be aged between three and 4.9 years, and not have any significant physical or intellectual disability which restricted them from participating in the intervention or impair the accuracy of physical activity measurement. Families meeting the inclusion criteria in each participating children’s centre were invited to take part in the project (n = 182). The final recruited sample consisted of seventy-seven families and seventy-nine children (mean age 3.7years, SD = 0.6; 51.9% male), equating to a 42% response rate. Subsequently, children’s centres were randomly allocated to either the intervention (n = 4) or comparison group (n = 4). Once the children’s centres were randomly allocated to their group, schedules for data collection and intervention delivery were devised. At post-test, the intervention and comparison group lost 1 and 2 families, respectively. Reasons for losses included moving house (n = 1) and time constraints (n = 2). The flow of participants through the study is illustrated in Figure 1[32]. The study was approved by the University ethics committee.

### Intervention design

This cluster randomised controlled trial was conducted for 10 weeks during the school autumn term (September to December 2011). The research design was implemented to avoid contamination across settings [33]. The 10 week duration was selected to fit the local authority school calendar and represented a significant period for observing short-term experimental effects. Assessments were conducted at baseline and immediately following the intervention.

### Intervention

#### Theoretical model

The intervention was designed using a socio-ecological model [34] and aimed to influence children’s total physical activity and time spent in sedentary behaviour. This was achieved by manipulating known mediators and moderators in the social environment [24,35]. Specifically, the intervention targeted parents as a key agent for physical activity promotion. The Foresight report [36] and the World Health Organisation [35] have indicated that a whole system approach to tackling behaviour change is critical, and have stressed the importance of considering behaviour change alongside environmental, policy and community approaches.

### Intervention: active play and parent’s educational workshops

The intervention followed the model recommended for developing and evaluating complex interventions [37]. Firstly, a user group was consulted on both the content and duration of the intervention. The use of such a group has been endorsed as it likely to result in better, more relevant science and a higher chance of producing implementable data [37]. The user group (n = 12) consisted of a convenience sample of parents, play workers, teachers and health promotion workers from within the children’s centre setting. Informal discussions were held with each user group member separately and notes were taken by the lead researcher. Once meetings with user group members were completed notes were shared with participants to check for accuracy. A draft intervention programme was then written using evidence from the literature combined with user group views. These were then supplemented by resources from programmes that targeted preschool children such as; Munch and Move [38,39], Unplug and Play [40], Change for Life [41], Free Range Kids [42], and Get Kids on the Go! [43]. The first draft of the intervention was then shared with an expert group (n = 5) including physical activity experts, paediatric exercise science researchers, a chartered sports psychologist and researchers working with parents on a local childhood obesity treatment programme. An overview of intervention content and associated components can be found in the supplementary material.

The intervention occurred every other week and comprised of 5 contact sessions over a 10 week period. Each session lasted approximately 70 minutes which consisted of 10 minutes registration and checking home activity completion and 60 minutes delivery time. Parents and children were separated for the first 20 of the 60 minutes. During this time the children participated in active play and the parents attended an educational workshop. The remaining 40 minutes of delivery was spent as one group participating in active play. The active play element of the intervention was delivered by team of professional play workers. The educational component of the parent’s workshops was delivered by the lead researcher and a research assistant who had previously worked on interventions targeting family behaviour change. Details of the intervention can be found in Additional file 1.

### Intervention implementation strategies

#### Move It! Snap It! Log It! Diary

On the first day of the intervention each family received a log book. The log book was adapted from one developed with families involved in a local child weight management programme [25]. The log book was one of a number of behaviour change techniques used within the intervention. Log books allowed families to self-monitor their home activity; permitted the research team to set graded tasks and provide instruction for these tasks; provide feedback on performance of the tasks; provide contingent rewards and allowed the families to agree to a behavioural contract [35]. Previous research supports the inclusion of self-monitoring of behaviour to prompt intention formation specific goal setting, providing feedback on performance, and prompting review of behavioural goals in interventions designed to promote physical activity [35,44]. Families were asked to bring their log book to each intervention session where they were reviewed by a member of the delivery team. Completed log books were linked to a progressive reward system. Rewards were linked to physical activity promotion such as activity bags, an Active Play key fob and an active dance DVD. Log books also contained contact details for additional support. Additionally, after completion of all post-test data collection, families in both the intervention and comparison group received a certificate, active play key fob, a Liverpool’s Little Stars activity song book and a £10 shopping voucher. The voucher was only rewarded if the families complied with all measurements.

### Provision of resources and instructional materials

Providing parents with instructional and educational material has been associated with positive changes in physical activity within this age group [45]. All families received resources and instructional materials throughout the intervention to allow them to implement the intervention at home and complete their home activities. The resources included current UK physical activity guidelines for the early years [46], Munch and Move fundamental movement skills teaching manual and accompanying games which encourage the development of such skills [47], Play4Life indoor and outdoor games ideas [48], a local active parks map, the British Heart Foundations ‘Get Kids on the Go’ activity booklet [49], local swimming pool schedules, 100 ways to Unplug n’ Play, Unplug n’ Play electronic media tally template, and Unplug n’ Play tips for setting family rules around screen time [50]. At the first session, all families were instructed to sign up for the Change4Life campaign [48].

### Follow up support

Follow up support can contribute to the effectiveness of an intervention [44,51]. During discussions with user group members it was evident that text messages were the most popular way (in comparison to phone calls, social media websites or email) to communicate key messages and contact families taking part in the intervention. Families received five text messages between each intervention session. Text messages were also used during the data collection weeks when families wore the accelerometer.

### Comparison group

Children’s centres allocated to the comparison group did not receive any intervention or associated materials during the study period. They were asked to continue with their usual physical activity provision and maintain their standard relationship with parents.

### Instrumentation and procedure

At baseline (0 weeks) and post-intervention (10 weeks) child and parent habitual physical activity was measured. At baseline the primary caregiver also completed the Pre-PAQ [31], detailed below.

### Children’s habitual physical activity

Physical activity was measured using 5 second epoch over 7 consecutive days (GT1M ActiGraph Pensacola, FL.). Participants were instructed to wear the accelerometers on an elastic belt on the right hip (anterior to the iliac crest). Parents were provided with a chart to document when the child put the monitor on and when it was taken off. This method of quantifying sedentary time and activity levels has been validated against direct observation in preschool aged children [52,53].

### Data management

MAHUffe (Analyser v 1.9.0.3) was used to analyse accelerometer data. Age specific cut-points were used to determine time spent sedentary or participating in light, moderate or vigorous physical activity [53]. Periods of 20 minutes of consecutive zeros were removed from the data as these were considered periods of non-wear time [54]. To be included as a valid measurement day, the accelerometer was required to be worn for a minimum amount of time during weekdays and weekend days. Wear times were calculated by defining 80% of the total length of time during which 70% of the sample wore the accelerometer [55]. This cut-off at baseline was 521 and 483 minutes for weekdays and weekend days, respectively and 466 and 448 min at post-test for weekdays and weekend days, respectively. Children were finally included if they wore the monitor for a minimum of 3 days including one weekend day [56,57].

### Adults habitual physical activity

Parent’s physical activity data was measured using the same accelerometer procedures as children. ActiGraph count cut-points for sedentary time (100cpm), light (≤1952cpm), moderate (≤5724cpm), and vigorous (>5725cpm) intensity physical activity [58] were used to determine parental sedentary time and physical activity levels. Periods of time greater than 60 minutes of consecutive zeros were considered periods of non-wear time and were not included in further analysis [59]. Minimum accelerometer wear time was calculated separately for weekdays and weekend days at baseline and post-test. This minimum wear time at baseline was 541 and 563 minutes for weekdays and weekend days, respectively and 602 and 500 minutes at post-test for weekdays and weekend days, respectively. Days during which participants did not achieve the minimal wear time were considered as non-compliant days and were not used in the analyses. Parents were included if they had 4 valid days of data including one weekend day [59]. Parents were classed as sufficiently active or insufficiently active depending on whether or not they achieved 30 minutes of moderate-to-vigorous physical activity on 5 days of the week [60].

### Questionnaire

A shorter version of the Pre-PAQ was administered to all parents before the intervention commenced. This tool has acceptable validity and reliability in this population [31]. Questions in reference to the child enrolled in the programme were completed by the parent. Parents were asked to proxy-report general information about their family unit, home and community environment, specific information surrounding the physical activity habits of themselves and their child participating in the programme. In section 1 (items 1–9) parents reported their relationship to the child, their age (years and months), current marital status, education level, ethnicity and the number of children living in the household. In section 2 (items 10–17) parents reported their full home postcode which was used to establish socio-economic status [30], the size of the area within their home perimeter, the availability of specific equipment within their home and backyard, the availability of specific electronic media within their home, available internet connection and the presence of a television in their child’s bedroom. Parents reported the presence of specific facilities in their neighbourhood, the amount of time their child spent in a car over the previous week (for weekday and weekend days) and the number of days their child actively travelled around their neighbourhood within the last week. In section 3 (items 19–25) parents reported the type of childcare and any organised activity their child attended in the last week. Finally, parents reported if their child usually consumed meals in front of the television. In section 4 (items 26–27) parents reported whether they had ever played sport at a competitive level and the nature of this sport.

### Data analysis

#### Exploratory analysis

Analyses were performed on an intention to treat basis. Full data (parent and child physical activity and questionnaire) were obtained for 58 families (32 comparison and 26 intervention) and used in subsequent analysis. Reasons for missing data included non-compliance with accelerometer procedure (n = 14), withdrawal from the study (n = 3) and loss of accelerometers (n = 4). Descriptive statistics were calculated to describe the final sample (Table 1). Independent t-tests were conducted to examine differences between participants who were either included or excluded in the physical activity analyses. The alpha level was set at *p* ≤ 0.05.

**Table 1:** **Baseline descriptive data (mean(SD))**

***Parents***	
Age (years)	33.7 (5.3)
% Male	26.6
% white British	91.2
Education	
% High school or less	63.4
% Technical or trade school	3.3
% University	33.3
% Married	70.7%
Minutes of weekday sedentary time	557.1 (127.3)
Minutes of weekend sedentary time	562.7 (168.4)
Minutes of weekday MVPA	42.6 (31.0)
Minutes of weekday MVPA	25.9 (28.8)
% achieving PA recommendations*	31.6%
***Children***	
Age (years)	3.8 (0.6)
% Male	51.9%
Minutes of weekday sedentary time	541.0 (77.1)
Minutes of weekend sedentary time	560.2 (80.5)
Minutes of weekday total physical activity	113.2 (24.9)
Minutes of weekday total physical activity	101.58 (30.1)
% achieving PA recommendations*	23.2 %

MVPA = moderate-to-vigorous physical activity.

* based on whole week physical activity.

### Main analysis – identifying significant predictor variables

A Pearson product moment correlation matrix was generated to assess correlation coefficients between the outcome variables and other confounding variables. Additionally, a stepwise backward regression was performed for each of the outcome variables to determine which variables best predicted the outcome. These data were analysed using PASW Statistics v.18, and the significance level was set at *p* ≤ 0.05.

To determine significant predictor variables multi-level modelling was conducted, which was considered the most appropriate technique for nested data [61]. A two-level data structure was used, where children were defined as the first level and school as the second level [62]. Data were analysed using MLwiN v.2.23 software (Centre for Multilevel Modelling, University of Bristol, UK). An association model was used to assess the effects of the predictor variables on the main outcome measures. Variables were added to the model in three stages [63] (1) significant variables identified in the backwards stepwise regression, (2) significant variables identified from the Pearson product moment correlation matrix, and (3) using empirical research to identify potentially confounding variables [15,17,64]. The sequence in which the predictor variables were added to the model can be found in the supplementary material. Please refer to Additional files 2 and 3. The effect of the predictor variables on the outcome variable was assessed for significance by comparing the −2 log likelihood (2*LL) for each model using the Chi-square distribution with 2 degrees of freedom and the Wald statistic. Alpha was set at *p* < .05 for all analyses [61].

### Main analysis – testing the intervention effect

Once all significant predictor variables for each of the four outcome variables were identified, the effect of the intervention was analysed using a three-level data structure. The three levels of analysis were time point (level one), child (level 2) and school (level 3). An association model was used to identify the effect of the intervention after being corrected for significant confounding variables. Two analyses were conducted on all four outcome variables (weekday sedentary time and total physical activity and weekend sedentary time and total physical activity) to examine the intervention effect over two time points. The first analysis (crude analysis) determined the effect of the intervention over time whilst controlling for baseline sedentary time or total physical activity, whilst the second analysis (adjusted analysis) determined the intervention effect when the covariates previously identified as significant predictor variables in the association model were added to the model [61]. In addition, potential effect modification was assessed by constructing interaction terms between the intervention group and all covariates. Separate analyses were conducted for weekday and weekend sedentary time and total physical activity. Regression coefficients in the model were assessed for significance using the Wald statistic [61]. Statistical significance was set at p < 0.05, with the exception of p < 0.1 which was used for interaction terms. Please refer to Additional file 4.

## Results

### Exploratory analysis

Independent samples t-tests revealed no statistically significant differences in sedentary time and total physical activity between boys and girls, between those who remained in the study and those who dropped out or between children with complete and incomplete physical activity data (p > 0.05). The accelerometer data showed that boys and girls engaged in 542.1 (64.7) and 545.3 (74.5) minutes of sedentary time during the weekday, respectively and 504.5 (99.1) and 510.4 (45.9) minutes of sedentary time during the weekend, respectively. Boys and girls engaged in 115.9 (21.4) and 110.1 (28.1) minutes of total physical activity during the weekday, respectively and 107.5 (29.7) and 97.0 (30.6) minutes of total physical activity during the weekend, respectively. The descriptive data for parents and children at baseline are displayed in Table 1. Independent-samples t-tests revealed that there were no significant differences between boys and girls or mothers and fathers in the intervention and comparison groups for age (*p* > 0.05). Ninety-one per cent of the sample was White British.

### Main analyses

Table 2 shows the effect of the intervention on sedentary time during weekdays and weekend days immediately after the intervention was delivered (10 weeks). A significant intervention effect was found for weekday and weekend day sedentary time. Children in the intervention group participated in 8.76 minutes (CI: -12.32 to −5.2) and 23.11 (CI: -29.17 to −17.06) less sedentary time during weekday and weekend days, respectively. When the correction for potential confounders was performed (adjusted analysis), the analysis revealed that parents participation in sport and child’s sex were significant predictors of weekday sedentary time. Further, data indicated that children whose parents previously participated in sport engaged in 7.12 minutes less sedentary time (CI: -9.57 to −4.67) than children whose parents were not regular sports participants. In terms of gender differences, girls engaged in 9.48 minutes more sedentary time (CI: 6.37 to 12.59) than boys. The number of television sets in the home, parents achieving the physical activity recommendations and child’s participation in organised sport were significant predictors of weekend sedentary time. Children who had less than the average number of televisions (3.06) at home accumulated 9.65 minutes less sedentary time (−14.84 to −4.46), while children whose parents achieved the physical activity recommendations accumulated 11.49 minutes less sedentary time (−13.99 to −8.99) and children who attended organised sport participated in 11.08 minutes less sedentary time (−19.01 to −3.15). All other covariates were not significant predictors of sedentary time; however they did improve the fit of the model and were therefore retained.

**Table 2:** **Estimated effects of covariateand intervention on sedentarytime during the weekand weekend days**

	** Weekday sedentary time**				** Weekend sedentary time**		
	**Model 1**	**Model 2**			**Model 1**	**Model 2**	
**Correlate**	***β*(SE**)	***β*(SE**)	**95% CI**	**Correlate**	***β*(SE**)	***β*(SE**)	**95% CI**
Constant	553.32 (6.04)	**569**.**39** (**23**.**66**)	**529**.**02 to 615**.**76**	Constant	517.85 (7.43)	**530**.**98** (**20**.**45**)	**490**.**85 to 571**.**01**
Intervention	−12.86 (8.55)	**−8**.**76** (**1**.**82**)	**−12**.**32 to −5**.**2**	Intervention	−1.82 (1.01)	**−23**.**11** (**3**.**09**)	**−29**.**17 to −17**.**06**
Attend organised activities		−5.67 (10.18)	−25.62 to 14.28	Minutes in car (weekend)		−0.15 (0.18)	−0.5 to 0.53
Parent’s play sport		**−7**.**12** (**1**.**32**)	**−9**.**57 to −4**.**67**	Number of TV’s at home		**−9**.**65** (**2**.**65**)	**−14**.**84 to** −**4**.**46**
Space to ride bike at home		−13.72 (16.72)	−46.49 to 19.05	Parent’s physical activity		−**11**.**49** (**1**.**28**)	−**13**.**99 to** −**8**.**99**
Number PC’s in home		1.13 (7.39)	−13.25 to 15.61	Parent’s play sport		−2.19 (3.14)	−8.34 to 3.96
Child’s sex		**9**.**48** (**1**.**59**)	**6**.**37 to 12**.**59**	Child’s age		−4.44 (10.74)	−24.49 to 16.61
Child’s age		9.01 (11.12)	−12.78 to 30.08	Number of sibling’s		−3.31 (10.28)	−23.45 to 16.83
TV in bedroom		12.81 (14.11)	−14.84 to 40.46	Attend organised activities		−**11**.**08** (**4**.**05**)	−**19**.**01 to** −**3**.**15**
Type of childcare attended		0.80 (2.99)	−4.88 to 6.48	Type of childcare attended		−1.57 (3.60)	−8.62 to 5.48
Neighbourhood playground		34.39 (23.61)	−11.88 to 58.00				
Neighbourhood pool		12.90 (13.47)	−7.5 to 33.3				
Neighbourhood gym		48.85 (51.59)	−52.26 to 149.96				
Play equipment at home		19.10 (17.31)	−14.82 to 53.02				
Number of TV’s at home		14.64 (8.05)	−1.13 to 30.41				
Internet at home		25.96 (32.53)	−43.97 to 102.16				
**Random**				**Random**			
School Level	0.00 (0.00)	32.60 (79.49)		School Level	0.00 (0.00)	28.97 (9.32)	
Child Level	1596.71 (239.36)	639.39 (144.01)		Child Level	1759.70 (287.65)	508.71 (93.23)	
Time point level	0.00 (0.00)	0.00 (0.00)		Time point level	0.00 (0.00)	0.00 (0.00)	
Deviance	909.009	467.193		Deviance	732.065	303.865	

Note: Significant effects are indicated in bold: * P ≤ .05, **P ≤ .01, ***P ≤ .001. Reference categories for intervention is comparison; for attend organised activities is no attendance; for parents participate in sport is no participation; for space to ride bike at home is ample space; for sex is boys; for neighbourhood playground is no playground; for neighbourhood pool is no pool; for neighbourhood gym is no gym; for play equipment at home is ample equipment; for internet at home is yes connection in place; for parents achieve physical activity recommendations is not achieved. Number PCs in home.

Child’s age, number of TV’s and PC’s at home, type of childcare attended, minutes in car (weekend) and number of siblings are reported as continuous variables where the average is centred around the grand mean (GM). The intervention ***β*** value represents the estimated difference in levels of sedentary time for the intervention centres against the comparison centres when all other parameters are included in the final model.

Abbreviations: *β* = Regression coefficient; *SE* = Standard Error; *CI* = Confidence Interval.

Table 3 shows the effect of the intervention on total physical activity during weekdays and weekend days immediately after the intervention was delivered (10 weeks). A significant intervention effect was found for weekday and weekend day total physical activity. Children in the intervention group participated in 4.70 (CI: 2.96 to 9.44) and 10.24 (CI: 10.24 to 18.08) minutes more physical activity than children in the comparison group during the weekday and weekend day, respectively. The results indicated that children of parents who participate in sport accumulated 4.54 (CI: 1.32 to 7.13) minutes more total physical activity than children whose parents do not. Parents who were sufficiently active i.e. they achieved the recommended 30 minutes per day of moderate-to-vigorous physical activity on 5 days of the week [60] were significant predictors of weekend total physical activity; children of parents who were more active participated in 9.08 (CI: 0.05 to 18.11) minutes more total activity than their non-active counterparts. All other covariates were not significant predictors of total physical activity; however they did improve the fit of the model and were therefore retained.

**Table 3:** **Estimated effects of covariateand intervention on totalphysical activity during theweek and weekend days**

	**Weekday Total Physical Activity**			**Weekend Total Physical Activity**			
	**Model 1**	**Model 2**			**Model 1**	**Model 2**	
**Correlate**	***β*** (**SE**)	***β*** (**SE**)	**95% CI**	**Correlate**	***β*** (**SE**)	***β*** (**SE**)	**95% CI**
Constant	107.99 (2.98)	**103**.**45** (**8**.**54**)	**86**.**71 to 120**.**18**	Constant	95.57 (3.35)	**78**.**27** (**9**.**39**)	**59**.**87 to 96**.**67**
Intervention	−0.97 (3.95)	**4**.**70** (**0**.**89**)	**2**.**96 to 9**.**44**	Intervention	2.48 (1.52)	**10**.**24** (**4**.**00**)	**2**.**4 to 18**.**08**
Parent’s play sport		**4**.**54** (**1**.**32**)	**1**.**95 to 7**.**13**	Parent’s physical activity		**9**.**08** (**4**.**61**)	**0**.**05 to 18**.**11**
Type of childcare attended		1.16 (1.21)	−1.21 to 3.53	Parent’s play sport		0.81 (4.86)	−8.72 to 10.34
Neighbourhood pool		5.27 (5.64)	−5.78 to 16.32	Space to ride bike at home		−6.81 (5.63)	−12.44 to 4.22
Parent’s sex		0.92 (5.54)	−9.93 to 11.77	Eat meals at TV		11.28 (7.51)	−3.43 to 25.99
				Minutes in car (weekday)		−0.06 (0.04)	−0.13 to 0.01
**Random**				**Random**			
School Level	40.49 (29.79)	36.53 (31.27)		School Level	0.00 (0.00)	371.95 (167.44)	
Child Level	218.44 (35.74)	200.12 (38.16)		Child Level	359.89 (60.40)	125.21 (38.68)	
Time point level	0.00 (0.00)	0.00 (0.00)		Time point level	0.00 (0.00)	0.00 (0.00)	
Deviance	742.666	570.172		Deviance	619.382	295.867	

Note: Significant effects are indicated in bold: * P ≤ .05, **P ≤ .01, ***P ≤ .001. Reference categories for intervention is comparison; for parents participate in sport is no participation; for neighbourhood pool is no pool; for parents gender is male; for parents achieve physical activity recommendations is not achieved; for space to ride bike at home is ample space; for eat meals at TV is does not eat at TV. Type of childcare attended and minutes in car (weekday) are reported as continuous variables where the average is centred on the grand mean (GM). The intervention β value represents the estimated difference in levels of sedentary time for the intervention centres against the comparison centres when all other parameters are included in the final model.

Abbreviations: *β* = Regression coefficient; *SE* = Standard Error; *CI* = Confidence Interval.

Potential effect modification resulted in a positive interaction term between the intervention and parents participation in sport (p < 0.10). There were no other significant interactions (see supplementary material).

## Discussion

The aim of this study was to investigate the effect of a 10-week family focused ‘Active Play’ intervention on children’s weekday and weekend day sedentary time and total physical activity. Secondary objectives were to investigate the influence of specific confounding variables on children’s weekday and weekend day sedentary time and total physical activity.

Compared with an age-matched comparison group, a family focused intervention delivered in children’s centres located in areas of high deprivation resulted in a positive significant intervention effect on children’s sedentary time and total physical activity assessed using accelerometry for weekday and weekend day. The presence of a significant intervention effect on children’s sedentary time and physical activity are similar to the findings from other empirical family focused studies, which have demonstrated significant increases in physical activity levels [65-67]. Furthermore these results suggest that children in the intervention group engaged in 1.5% and 4.3% less sedentary time during weekdays and weekend days respectively, and 4.5% and 13.1% more total physical activity during weekdays and weekend days respectively than children in the comparison group. Of interest is the change in sedentary time and physical activity from weekdays to weekend days. The results indicate that children in the intervention group participated in 23.1 minutes less sedentary time and 10.2 minutes more total physical activity than children in the comparison group. If maintained, this equates to approximately 64 hours less sedentary time and 16 hours more total physical activity over 6 months, which in turn may have positive effects on children’s BMI [68], cardio metabolic disease [2] and fundamental movement skills [69]. A possible reason for this may be that children were more exposed to support from their parents at the weekend which is positively associated with children’s physical activity at home but not when attending childcare [28]. The positive changes in children’s sedentary time and physical activity suggest that the intervention successfully convinced parents about the importance of physical activity for their children. Further, our findings confirmed that parents were motivated to encourage their children to spend more time engaging in physical activity and less time in sedentary behaviours. The intervention influenced factual and direct messages that matched the preferences of parents with young children. The varied conveyance of key messages to parents during the intervention allowed for differences between parents’ knowledge base and their ability to process information e.g. through practical tasks, group discussion, supplementary information and text alerts [21].

Compared with other interventions varying in duration from six months to three years [2,65,70-72], this intervention was relatively short in duration, with contact sessions occurring every other week. The significant reduction in sedentary time and increase in total physical activity may be attributed to the intense delivery style, continual reinforcement of key messages and active involvement of parents over the 10 weeks [2,45,65]. Parents and children received high exposure [73] to the intervention, for example both participating in the Active Play sessions together, which has been found to positively affect changes in behaviour over time [74]. To maximise the chances of a long term intervention effect we employed a number of behaviour change processes and techniques. Similar to other studies [65], these included building self-efficacy by setting home activities and providing performance feedback, identifying and motivating readiness to change by consistently providing general information on the importance of physical activity for young children. Follow up prompts were also used in the prevention and management of relapse, this included sending text alerts with key messages relating to home-based activity. Parents were asked to log their home activity progress in the “Move It, Snap It, Log It” diary. Process evaluation at post-intervention implied that parents had increased their awareness of the importance of physical activity and made behavioural changes. While this is a promising indicator of the intervention effect, this information told us little about the short or long-term changes made by the families and whether these behaviours had become habitual. Our intervention also placed a strong emphasis on parental role-modelling, with parents encouraged to join in the active play sessions; complete the home activity diary with their child and attend the end of intervention celebration event together.

A review of the correlates of sedentary time [17] and physical activity [15] in preschool children highlight how these behaviours are influenced by individual and environmental factors. In this study, a number of confounders for weekday and weekend day sedentary time and total physical activity were identified. These included parent’s participation in sport and their physical activity levels, child’s sex, availability of media in the home and attendance at organised activities. Potential effect modification was assessed for all covariates in order to investigate whether the intervention effect was different for different subgroups [61]. The results revealed a significant interaction for parent’s participation in sport, but not for any other variables. The intervention effect was stronger for weekday physical activity for children whose parents participated in sport. This finding may be related to the positive relationship which exists between increased child activity and parents own activity levels as well as their support for their child’s physical activity [28].

Gustafson and colleagues [22] conducted a review on the parental correlates of children’s physical activity and despite a lack of existing studies to draw firm conclusions from; unanimous results supported the importance of parents’ physical activity on their children’s activity levels. In the current study parent’s participation in sport and physical activity were positively associated with children’s physical activity levels and sedentary time. Few studies have investigated the relationship between parent and child activity levels among children in this age group using an objective measure of physical activity; research using self-report as a measure of physical activity for parents report conflicting results ranging from no relationship with accelerometer-derived physical activity [3] to positive results with directly observed physical activity [75,76]. Other studies which have objectively monitored parent’s physical activity have also reported a significant positive association between parent and child levels of activity [24,77]. This study adds objective evidence for a relationship between parent’s activity and child’s sedentary time, highlighting the importance of parental involvement in preschool physical activity intervention design and promotion. It is difficult to state the precise nature of parental involvement required. Our results suggest that parents should be encouraged to be physically active themselves to stimulate increased child physical activity.

Previous family focused studies have evaluated the effects that enable children to be active, including providing a family orientated health education programme, as well as the provision of extra physical activity [78]. While some empirical research has compared intervention effects between boys and girls activity, to the best of the authors knowledge, no family focused intervention studies have considered the effect of the intervention effect or the differences in the intervention effect when individual and environmental factors have been controlled for.

Consistent with most other studies boys accumulated less sedentary time than girls during weekdays [64]. In contrast to our findings, a review of sedentary time correlates concluded that there was an indeterminate association between child’s sex and sedentary behaviour as measured by accelerometry [17]. The contrasting findings are perhaps due to the multi-dimensional nature of children’s sedentary behaviours and the lack of consistent evidence surrounding sedentary time and other potential correlates [17]. Other studies investigating the relationship between child’s sex and sedentary time have found inconsistent results [15,79,80]. We found no gender differences for physical activity; however we did not investigate intensity specific physical activity such as moderate and vigorous levels. The number of television sets in the child’s home significantly contributed to children’s sedentary time, no other studies report the number of televisions in the home, however television viewing and the presence of a television set in the home have been the most commonly examined sedentary behaviour, but a lack of consistency within studies make it difficult to draw robust conclusions about associations [17]. Lastly, children who attended organised activities accumulated less sedentary time at the weekend, this maybe also related to parents support for physical activity and their likelihood to facilitate engagement by participation in active play at home, by playing with their child, providing transportation to parks and other activity-related facilities, and providing reinforcement for physical activity participation [28].

Our study has several unique elements. First, our intervention moves beyond an educational focus by fostering a “learning by doing” approach evident within the child and parent Active Play sessions. Second, we have designed and implemented a multi-component intervention that incorporates an existing Active Play programme to promote physical activity in this age group. Third, this intervention was inexpensive and relatively straight forward to implement costing approximately £4.12 per family per week to deliver. As a fourth element we use a multi-pronged strategy to change behaviours. We chose to broaden our focus by including lifestyle-related activities (e.g. encouraging active travel) that could be practiced daily. We also included ‘non-sport related’ forms of physical activity (e.g. providing an interactive dance resource and a city map of green spaces and playgrounds), which may appeal to the broader preschool population and their families. Finally, the use of an objective measure of sedentary time and physical activity as well as the use of multilevel analyses adds to the rigour of our methodology.

Despite its strengths, we acknowledge the limitations of our study design. Our intervention does not target all levels of the socio-ecological system, in which pre-schoolers’ behaviours develop. For example, the intervention has not been developed with teachers and childcare staff in mind and is not anchored within the early year’s foundation stage national curriculum. Previous research suggests that this might hinder the readiness of teachers to take ownership in the intervention change process [81]. Second, while a user group was formed and its members consulted individually on the intervention content initially, they were not consulted on the planning of the intervention. Therefore, our study cannot purely be characterised as community-based research. However, a systematic review of community-based research found only 4 of 60 studies demonstrating community participation across all research phases [82]. A further limitation of our intervention is that due to time restraints the initial set of ideas was not refined and discussed with input from parents of children enrolled at the intervention preschools, but rather from parents involved in the user group. Our intervention required a degree of parental time commitment at a level that might exceed parental resources. This may, in turn, threaten sustainability through fluctuations in parental time availability and as children progress from voluntary childcare to mandatory formal preschool over the next 1–2 years. Future interventions should consider including preschool teachers in elements of interventions to assist with the adoption of key messages thus limiting potential effects on the changing school process. Additionally, there was a low number of fathers involved in the intervention, future studies should make an effort to involve more fathers given how influential their parenting styles can be on preschool children’s makers of health [83]. Lastly, the absence of a long term follow-up does not allow us to make concrete assumptions on the sustainability of the intervention.

## Conclusions

This investigation contributed to the dearth of empirical literature investigating the short-term effects of a family focused intervention on preschool children’s sedentary time and total physical activity. Our findings suggest that the effect of the intervention was significant in decreasing children’s sedentary time and increasing their physical activity. These findings are important from a health promotion perspective as they reiterate the importance of a family approach, by directly involving parents in the intervention programme. In this study, a significant interaction term indicated that the effects of the intervention were stronger for children whose parents participated in sport. This study also identified a number of confounding variables which have a significant effect on children’s sedentary time and total physical activity, with the most frequent confounding variable being parents own physical activity levels and their participation in sport. From an ecological perspective, the results suggest that children whose parents are sufficiently active and participate in sport, those with fewer televisions at home and attend organised activities are the children who are most likely to habitually participate in health enhancing physical activity. There is need to evaluate the longer-term effects of family focused physical activity interventions in this age group.

## Competing interests

The authors declare that they have no competing interests.

## Authors’ contributions

MVOD and GS conceived the study and secured funding. MVOD, SJF, ZK and GS contributed to the planning and design of the study. MVOD collected the data. MVOD and SJF conducted data manipulation and analyses. MVOD wrote the manuscript and SJF, ZK and GS supplied comments. All authors read and approved the final manuscript.

## Declaration of interest

This study formed part of Mareesa O’Dwyer’s doctoral programme of research and was funded by the Neighbourhood Renewal Fund, Liverpool Children’s Services and Liverpool John Moores University.

## Supplementary Material

Additional file 1 **Table S1.** Overview of intervention content. Click here for file

Additonal file 2 **Table S2.** Order of predictor variables entered into the sedentary time models. Click here for file

Additional file 3 **Table S3.** Order of predictor variables entered into the total physical activity time models. Click here for file

Additional file 4 **Table S4.** Intervention interaction terms with covariates investigating potential effect modification. Click here for file
