# Supplementary material for: Effect of a family focused active play intervention on sedentary time and physical activity in preschool children
Source: Int J Behav Nutr Phys Act. 2012 Oct 1;9:117. doi: 10.1186/1479-5868-9-117 (PMC3495835; doi:10.1186/1479-5868-9-117)
Supplement: Additional file 1 — Table S1. Overview of intervention content. [file 1479-5868-9-117-S1.docx]

**Table – supplementary 1** Overview of intervention content

|  | **Session theme & rationale** | **Active play delivery** | **Parent(s)**  **workshop element** | **Programme home linked activity** |
| --- | --- | --- | --- | --- |
| ***Session 1*** | ***Importance of involving parents & the new PA guidelines***  Start Active, Stay Active physical activity guidelines.  Parents unsure of physical activity guidelines.  Family-based interventions targeting preschoolers should include strategies to increase parental support for physical activity. | Introduction to active play: movement with confidence, spacial awareness and responding to activity based instructions. | Importance of parent(s) in physical activity promotion and behavior change  Introduction to new physical activity guidelines. | Allow child to choose one activity each day and play this with them for 10 minutes.  Sign up for Change4Life |
| ***Session 2*** | ***Stages of development & fundamental movement skills***  Proficient FMS during preschool years is correlated with increased PA levels.  Age 2 – 7: window of opportunity for skill development. | Explore balancing, hopping and throwing through different games. | Developmental stages of early childhood  What are FMS?  Why are FMS important? | Parent(s) and child are asked to perform a  locomotor, object control , balance or stability game each day for 10 minutes. |
| ***Session 3*** | ***Overcoming the barriers that exist in outdoor and indoor play. Let’s get risky!***  Parents do not want their children to play outdoors.  Outdoors associated with dangerous and taking risks. | Using an obstacle course, encourage parent(s) to take risks with their children whilst being active. | Discussions about societal changes. Breaking down the barriers. | Parent(s) and child are asked to take part in one indoor or one outdoor activity they usually don’t participate in each day for 10 minutes. |
|  |  |  |  |  |
| ***Session 4*** | ***Energetic play – (using P.O.W. message: Pulse higher – Out of breath – Warm feeling)***  Preschool children are not accumulating enough MVPA for health benefits.  MVPA is important for child development. | Using a variety of fun games, aim to get children ‘out of breath’ and red in the face. | Learn to identify different physical activity intensities  Discuss why high intensity physical activity is important? | Parent(s) and child take part in 10 minutes per day of high intensity activity  Self monitor child’s screen time using the chart provided. |
| ***Session 5*** | ***Reducing screen time & Celebration Event***  TV associated with obesity in preschool children.  Excessive TV in child care settings.  TV and irregular sleep patterns. | Main activity involved Musical Mats to Liverpool Little Stars activity song resource. | Define screen time and the guidelines  Discuss alternatives and ways to limit screen based activity. | Using the family rules template limit screen time each day. |
