# Supplementary material for: Effect of a family focused active play intervention on sedentary time and physical activity in preschool children
Source: Int J Behav Nutr Phys Act. 2012 Oct 1;9:117. doi: 10.1186/1479-5868-9-117 (PMC3495835; doi:10.1186/1479-5868-9-117)
Supplement: Additonal file 2 — Table S2. Order of predictor variables entered into the sedentary time models. [file 1479-5868-9-117-S2.docx]

**Table – supplementary 2** Order of predictor variables entered into the sedentary time models

| **Weekday sedentary time** | **Weekend sedentary time** |
| --- | --- |
| Time spent in car during weekend | **Time spent in car during weekend** |
| **Attend organised activities** | **Number of TVs at home** |
| Parent’s physical activity | **Parent’s physical activity** |
| **Parent’s play sport** | **Parent’s play sport** |
| **Space to ride bike at home** | Child’s sex |
| **Number of PC’s at home** | **Number of sibling’s** |
| Neighbourhood natural space | **Child’s age** |
| **Child’s sex** | **Attend organised activities** |
| **Childs age** | TV in bedroom |
| Number of sibling’s | Time spent in car during weekday |
| **TV in bedroom** | Number of day’s active travel per week |
| Time spent in car during week day | **Type of childcare attended** |
| Number of day’s active travel per week | Eat meals at TV |
| **Type of childcare attended** | Parent’s sex |
| Eat meals at TV | Parent’s age |
| Parent’s sex | Ethnicity |
| Parent’s age | Parent’s highest level of education |
| Ethnicity | Neighbourhood playground |
| Parent’s highest level of education | Neighbourhood green space |
| **Neighbourhood playground** | Neighbourhood park |
| Neighbourhood green space | Neighbourhood pool |
| Neighbourhood park | Neighbourhood gym |
| **Neighbourhood pool** | Neighbourhood natural space |
| **Neighbourhood gym** | Access to play equipment at home |
| **Access to play equipment at home** | Space to ride bike at home |
| Number of TV’s at home | Number of PC’s at home |
| **Internet access at home** | Internet access at home |
| Significant predictor variables are indicated in bold and were retained for the final association model | |
