# Supplementary material for: Effect of a family focused active play intervention on sedentary time and physical activity in preschool children
Source: Int J Behav Nutr Phys Act. 2012 Oct 1;9:117. doi: 10.1186/1479-5868-9-117 (PMC3495835; doi:10.1186/1479-5868-9-117)
Supplement: Additional file 3 — Table S3. Order of predictor variables entered into the total physical activity time models. [file 1479-5868-9-117-S3.docx]

**Table** – **supplementary 3** Order of predictor variables entered into the total physical activity time models

| **Weekday sedentary time** | **Weekend sedentary time** |
| --- | --- |
| Child’s age | Internet access at home |
| Ethnicity | Childs age |
| Parent’s physical activity | Ethnicity |
| **Parent’s play sport** | **Parent’s physical activity** |
| Child’s sex | **Parent’s play sport** |
| **Type of childcare attended** | Child’s sex |
| Space to ride bike at home | Type of childcare attended |
| Access to play equipment at home | **Space to ride bike at home** |
| Neighbourhood playground | Access to play equipment at home |
| Neighbourhood park | Neighbourhood playground |
| Neighbourhood green space | Neighbourhood park |
| **Neighbourhood pool** | Neighbourhood green space |
| Neighbourhood natural space | Neighbourhood pool |
| Neighbourhood gym | Neighbourhood natural space |
| Number of sibling’s | Neighbourhood gym |
| TV in bedroom | Attend organised activities |
| Number of TVs at home | TV in bedroom |
| Eat meals at TV | Number of TV’s at home |
| Time spent in car during weekday | **Eat meals at TV** |
| Time spent in car during weekend | **Time spent in car during weekday** |
| Number of day’s active travel | Time spent in car during weekend |
| Attend organised activities | Number of day’s active travel |
| Parent’s age | Parent’s age |
| **Parent’s sex** | Parent’s sex |
| Parent’s level of highest education | Parent’s level of highest education |
| Number of PC’s at home | Number of PC’s at home |
| Internet access at home | Number of sibling’s |
| Significant predictor variables are indicated in bold and were retained for the final association model | |
