# Supplementary material for: Effect of a family focused active play intervention on sedentary time and physical activity in preschool children
Source: Int J Behav Nutr Phys Act. 2012 Oct 1;9:117. doi: 10.1186/1479-5868-9-117 (PMC3495835; doi:10.1186/1479-5868-9-117)
Supplement: Additional file 4 — Table S4. Intervention interaction terms with covariates investigating potential effect modification. [file 1479-5868-9-117-S4.docx]

**Table – supplementary 4** Intervention interaction terms with covariates investigating potential effect modification

| **Interaction term** | **β** | **SE** | ***P*- value** |
| --- | --- | --- | --- |
| **Weekday sedentary time**  Intervention x attend organised activities  Intervention x parent’s play sport  Intervention x space to ride bike at home  Intervention x number PC’s in home  Intervention x sex  Intervention x child’s age  Intervention x TV in bedroom  Intervention x type of childcare attended  Intervention x neighbourhood playground  Intervention x neighbourhood pool  Intervention x neighbourhood gym  Intervention x number of TV’s at home  Intervention x internet at home | -17.13  -22.43  -18.17  -5.07  -2.63  -23.50  33.42  1.86  -12.11  -33.85  8.50  6.65  -56.69 | 19.63  18.20  20.47  12.72  17.00  15.20  20.28  5.70  18.51  32.09  11.19  9.89  49.56 | 0.68  0.46  0.67  0.92  0.99  0.32  0.25  0.94  0.80  0.57  0.74  0.79  0.51 |
| **Weekend sedentary time**  Intervention x minutes in car (weekend)  Intervention x number of TV’s at home  Intervention x parents physical activity  Intervention x parents play sport  Intervention x child’s age  Intervention x number of sibling’s  Intervention x attend organised activities  Intervention x type of childcare attended | 0.01  13.46  -28.77  -15.98  0.60  7.28  -22.37  1.13 | 0.28  11.89  23.23  16.41  6.29  11.98  18.52  7.53 | 1.00  0.52  0.46  0.62  0.99  0.83  0.48  0.99 |
| **Weekday total physical activity**  Intervention x parent’s play sport  Intervention x type of childcare attended  Intervention x neighbourhood pool  Intervention x parent’s sex | 14.23  0.26  2.66  4.09 | 5.32  2.23  11.04  7.90 | 0.07 *  0.99  0.97  0.87 |
| **Weekend total physical activity**  Intervention x parent’s physical activity  Intervention x parent’s play sport  Intervention x space to ride bike at home  Intervention x eat meals at TV  Intervention x minutes in car (weekday) | 1.47  2.04  2.91  -10.25  0.12 | 13.76  10.36  13.05  10.97  0.09 | 0.99  0.98  0.97  0.64  0.41 |
| * p < 0.10 | | | |
